# The association between the residential environment and arthritis among the elderly in China: evidence from cohort and cross-sectional studies

**DOI:** 10.7189/jogh.16.04276

**Published:** 2026-08-14

**Authors:** Xiangbin Jia, Xin Xiong, Zichao Jiang, Haoze Yang

**Affiliations:** 1 Center for Medical Genetics and Hunan Key Laboratory of Medical Genetics, School of Life Sciences, Central South University, Changsha, Hunan, China; 2 Department of Epidemiology and Health Statistics, Xiangya School of Public Health, Central South University, Changsha, China; 3 Department of Orthopaedic Surgery, The First Affiliated Hospital, College of Medicine, Zhejiang University, Hangzhou, Zhejiang, China; 4 Department of Anaesthesiology, The Second Xiangya Hospital of Central South University, Changsha, Hunan, China

**Keywords:** arthritis, residential environment, CLHLS, public health, cross-sectional study, cohort study

## Abstract

**Background:**

Arthritis, characterised by inflammation, pain, and joint stiffness, is emerging as a significant health concern in ageing populations worldwide. While many studies have examined the effects of outdoor environmental risk factors on arthritis, fewer have investigated the effects of residential ecological factors.

**Methods:**

We included 2,511 participants in the cohort study and 13,886 participants in the cross-sectional study from the Chinese Longitudinal Healthy Longevity Survey. We employed multivariate Cox proportional hazards and logistic regression to analyse the cohort and cross-sectional data. For each residential environment factor, we developed three models, with model three adjusted for all potential confounders collected in our study, which we considered our primary model.

**Results:**

We identified a significant association between the use of non-clean fuel and a higher risk of arthritis (hazard ratio (HR) = 1.42; 95% confidence interval (CI) = 1.05–1.93) through cohort analysis. Conversely, residing in high-rise buildings with elevators was associated with a reduced risk of arthritis (HR = 0.12; 95% CI = 0.02–0.86). Our cross-sectional study reflects a reduced risk of arthritis associated with summer window ventilation for one to five times/week (odds ratio (OR) = 0.70; 95% CI = 0.50–0.97) and more than five times/week (OR = 0.66; 95% CI = 0.48–0.90). Additionally, distance of >300 m was also associated with a lower risk of arthritis (OR = 0.79; 95% CI = 0.69–0.89).

**Conclusion:**

These findings underscore the crucial role of residential environmental factors in the risk of arthritis development. These results have important implications for public health policies aimed at reducing the prevalence of arthritis.

Arthritis is a long-term condition affecting the joints, characterised chiefly by pain, stiffness, and swelling [1,2]. Its severe condition can cause joint deformity, while functional limitation is also observed [3]. Common types include osteoarthritis and rheumatoid arthritis [4,5]. With the global ageing population, the prevalence of arthritis is steadily increasing, imposing a significant social and economic burden [6,7]. Arthritis is one of the leading causes of disability, severely impacting patients' quality of life [8,9]. The high prevalence of arthritis and its substantial societal burden underscore the need for such comprehensive investigations, particularly in the context of an ageing society [10–12].

Current research on arthritis risk factors has mainly focused on outdoor environmental factors, such as air pollution, temperature changes, and physical labour [13–15]. However, with the acceleration of urbanisation, people are spending more time indoors, making the impact of residential environments on health increasingly significant [16–18]. Some factors, such as residential air quality, living conditions, and household atmosphere, may also play crucial roles in the occurrence and progression of arthritis. However, studies on the relationship between residential environments and arthritis risk are relatively scarce, limiting our comprehensive understanding of these potential risk factors [19,20]. Biologically, poor indoor air quality, dampness, mould, and household fuel pollution may increase oxidative stress and low-grade systemic inflammation, both of which are involved in joint degeneration and inflammatory arthritis [16]. In addition, uncomfortable or stressful living conditions may affect sleep, physical activity, and immune regulation, which could further influence arthritis risk or symptom progression [21]. Therefore, the residential environment may not only reflect living conditions but also contribute to inflammatory and degenerative joint processes.

Therefore, conducting extensive research into the impact of residential environments on arthritis is crucial. This study utilises data from the Chinese Longitudinal Healthy Longevity Survey (CLHLS) to comprehensively explore the associations between residential environments and arthritis. We employed both cohort and cross-sectional study designs. The cohort analysis examined the long-term effects of residential environment factors on arthritis, while the cross-sectional analysis investigated associations with factors first studied in 2018. By integrating these approaches, we aimed to identify and validate a wide range of influences on arthritis incidence, with a particular focus on residential environmental factors. This research not only fills gaps in the current literature but also provides scientific evidence to inform the development of arthritis prevention and control strategies, holding significant theoretical and practical value.

## METHODS

### Study population

We followed the STROBE and GRABDROP reporting guidelines (Tables S1 and S2 in the **Online Supplementary Document**). CLHLS is a study conducted by Peking University’s Centre for Healthy Ageing and Development Studies to assess the health status of Chinese seniors and their related social, behavioural, and biological factors [22–24]. Participating in CLHLS are Chinese people aged ≥65 years from 23 provinces nationwide.

**Figure 1:**
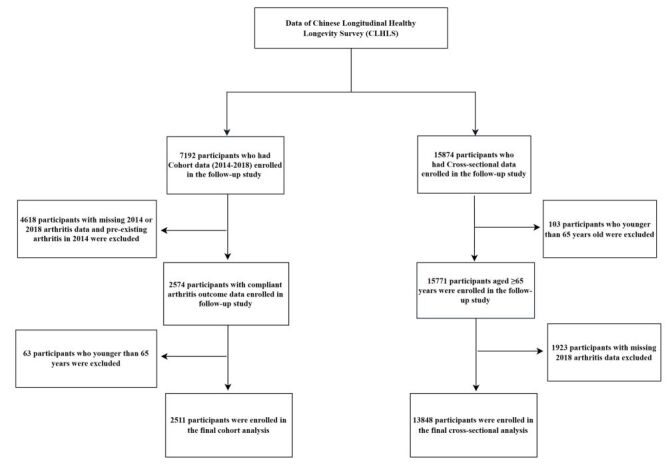
Flow diagram depicting participant recruitment and retention.

Residential environmental variables were assigned to the cohort or cross-sectional analyses according to their availability across survey waves. Variables measured at baseline and linked to follow-up arthritis status were included in the cohort analysis, whereas variables available only at the follow-up survey or without comparable baseline measurements were examined in the cross-sectional analysis. For our cohort study, we utilised the 2014–2018 data set from the CLHLS [24], which included 7,192 participants from two survey waves in 2014 and 2018. After excluding individuals who were under 65 years old, had missing data on the outcome (*i.e.* arthritis), or had a history of arthritis before the 2014 wave, we included a total of 2,511 participants (**Figure 1**). Regarding the cross-sectional study, we used the data set from the 2018 wave of the CLHLS, which included 15,874 participants. The same exclusion criteria were applied, resulting in a final sample of 13,886 participants.

### Exposures and outcome

Our outcome variable was arthritis, defined by the question, ‘Do you currently have arthritis?’

We included 12 residential environment factors as exposures, six from the cohort study and the others from a cross-sectional study. In the cohort study, six factors were water leakage in the past year (yes or no), musty smell (yes or no), fuel (clean fuel, including options: never cooking, gas, electricity, fuel oil, kerosene, and solar energy) or non-clean fuel (including options: firewood or straw, charcoal, and coal or coke), cohabitant (living with family, elderly care institutions, or alone), building type (low-rise apartment; <4 floors), high-rise without elevator (≥4 floors), or high-rise with elevator; ≥4 floors) and bedroom (separate or non-separate). Six variables in our cross-sectional study were: window ventilation in summer (zero time/week, one to five times/week or >5 times/week), window ventilation in winter (zero time/week, one to five times/week or >5 times/week), using insecticide (yes or no), using repellent (yes or no), using toilet cleaner (yes or no) and distance from the main road (0–100 m, 101–300meters, or >300 m).

### Covariates

We included a set of covariates in our cohort and cross-sectional studies, respectively, as potential confounders based on clinical practice, prior knowledge, and data availability. In the cohort study, covariates included age, gender (female or male), ethnicity (Han or non-Han), marital status (divorce/widowed, married, or unmarried), income (<10,000, <50,000, or ≥50,000 CNY/y), school years, residential area (city, town, or rural), and house ownership (yes or no). In the cross-sectional study, covariates included building type, fuel, cohabitant, mildew smell, and water leakage.

### Statistical analysis

#### Descriptive analysis

Outcomes and covariates were presented by outcome group. Using the Kolmogorov-Smirnov test, we assessed the normality of continuous variables, summarising normally distributed data with mean and standard deviation and non-normally distributed data with median and interquartile range (IQR). We expressed categorical data as frequencies and percentages. We compared groups using the Mann-Whitney test, *t* test, and χ^2^ test for non-normally distributed continuous, normally distributed continuous, and categorical data, respectively.

#### Multiple imputation

In the cohort and cross-sectional studies, 11 and 16 variables had missing values, respectively (Tables S3 and S4 in the **Online Supplementary Document**). Missing data in both studies were assumed to be missing at random and imputed into 15 and 20 data sets, respectively, using the multiple imputation by chained equations (MICE) method with the ‘mice’ package, version 3.16.0 in *R*, version 4.3.2 (R Foundation for Statistical Computing, Vienna, Austria). We performed all multivariate Cox and logistic regression analyses on each imputed data set, and pooled the regression coefficients using Rubin’s rules.

#### Multivariate Cox and logistic regression analysis

In our cohort study, we utilised Cox proportional hazards regression to investigate the health impact of residential environment factors on arthritis. We calculated hazard ratios (HRs) and their corresponding 95% confidence intervals (CIs) to estimate the effect. The proportional hazards assumption was tested using the Schoenfeld residuals method, with *P* < 0.05 indicating a violation [25]. Follow-up time was defined as the duration between the date of the 2014 and the 2018 wave surveys. We developed three models for each outcome, each specified as outcome–exposure plus confounders. Model one was the unadjusted model (*i.e.* without any confounder included). Model two was adjusted for age, gender, ethnicity, income, school years and marital status. Model three was adjusted separately for each exposure’s potential confounders, making it the primary model for providing more credible estimates of these associations. The proportional hazards assumption was assessed using Schoenfeld residuals for the fully adjusted Cox model (model three) of each exposure. The results indicated that the assumption was generally satisfied for most residential environmental exposures, whereas water leakage showed evidence of violation of the proportional hazards assumption. Therefore, according to the recommendations of Stensrud et al. [26], the hazard ratio estimate for water leakage should be interpreted as a weighted average of the underlying time-varying hazard ratios over the entire follow-up period. For the cross-sectional study, we used logistic regression to explore the association between exposures and arthritis. Adjusted odds ratios (ORs) and their corresponding 95% CIs were calculated to estimate the associations. The same criteria applied in the cohort study were used to develop three models in this study (Table S5 in the **Online Supplementary Document**)

For both cohort and cross-sectional studies, we used only complete cases to assess the robustness of our results.

## RESULTS

### Cohort study

**Figure 2:**
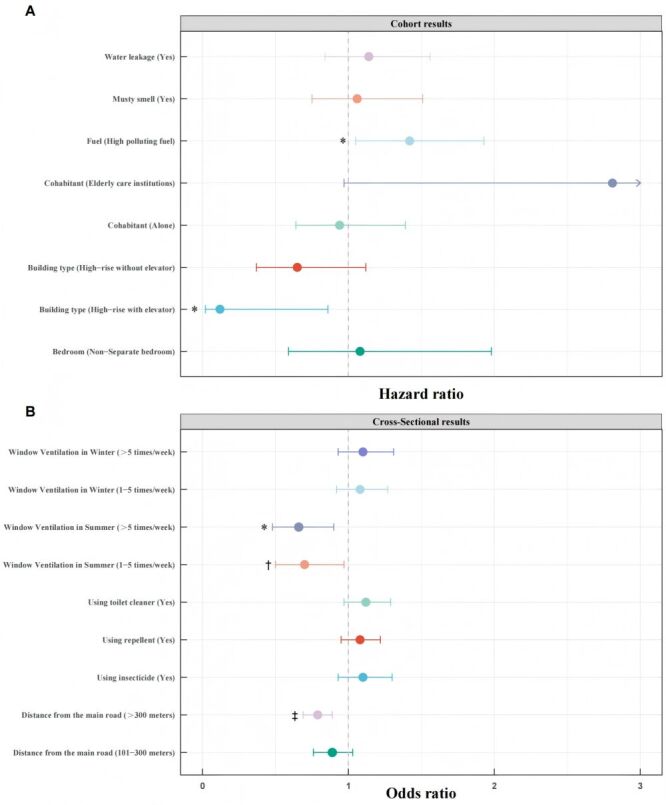
Forest map of the association between residential environment factors and arthritis. Panel A. Results of cohort study. Panel B. Results of cross-sectional study. *P < 0.05, †*P* < 0.01, ‡*P* < 0.001.

We included 2511 elderly individuals in the study, of whom 228 (9.1%) developed arthritis (**Table 1**). The median age of the participants was 80.0 years (IQR = 75.0–88.0), and nearly half of them were female (50.6%). Notably, the proportion of females among participants with arthritis (61.8%) was significantly higher than among those without arthritis (49.5%). We found that elderly individuals using highly polluting fuel had a higher risk of developing arthritis (HR = 1.42; 95% CI = 1.05–1.93). Conversely, individuals living in high-rise buildings with elevators had a lower risk of developing arthritis (HR = 0.12; 95% CI = 0.02–0.86) (**Figure 2**, Panel A, **Table 2**).

**Table 1:** Basic characteristics of the cohort study population stratified by arthritis*

Characteristics	All	Without arthritis	Arthritis
Age in years, Mdn (IQR)	80.0 (75.0–88.0)	81.0 (75.0–88.0)	79.0 (74.0–87.0)
Gender (female)	1,271 (50.6)	1,130 (49.5)	141 (61.8)
Marital status			
*Divorce/widowed*	1,200 (48.5)	1,092 (48.5)	108 (48.2)
*Married*	1,248 (50.4)	1,137 (50.5)	111 (49.6)
*Unmarried*	28 (1.13)	23 (1.02)	5 (2.23)
Income in CNY per year			
*<10,000*	825 (35.0)	748 (34.8)	77 (37.7)
*<50,000*	1,023 (43.5)	944 (43.9)	79 (38.7)
*≥50,000*	506 (21.5)	458 (21.3)	48 (23.5)
School years, Mdn (IQR)	0.00 (0.00–5.00)	0.00 (0.00–5.00)	0.00 (0.00–5.00)
Residential area			
*City*	256 (10.2)	220 (9.6)	36 (15.8)
*Town*	755 (30.1)	696 (30.5)	59 (25.9)
*Rural*	1,500 (59.7)	1,367 (59.9)	133 (58.3)
Ethnicity (Han)	2,047 (92.1)	1,848 (91.9)	199 (93.9)
House ownership (yes)	2,366 (95.0)	2,153 (95.0)	213 (95.1)
Cohabitant			
*Living with family*	1,925 (77.4)	1,755 (77.6)	170 (75.2)
*Alone*	543 (21.8)	491 (21.7)	52 (23.0)
*Old people institution*	19 (0.7)	15 (0.6)	4 (1.7)
Bedroom (separate bedroom)	2,352 (94.7)	2,143 (94.7)	209 (94.6)
Building type			
*Low-rise apartment (<4 floors)*	2,193 (88.3)	2,001 (88.5)	192 (86.1)
*High-rise without elevator (≥4 floors)*	249 (10.0)	220 (9.7)	29 (13.0)
*High rise with elevator*	41 (1.6)	39 (1.7)	2 (0.9)
Water leakage (yes)	519 (20.9)	462 (20.5)	57 (25.4)
Musty smell (yes)	386 (15.5)	344 (15.2)	42 (18.8)
Fuel (clean)	1391 (55.8)	1,272 (56.1)	119 (53.4)
Follow-up time, Mdn (IQR)	1,441 (1,267–1,556)	1,437 (1,266–1,556)	1,500 (1,290–1,562)

IQR – interquartile range, Mdn – median

*Presented as n (%) unless specified otherwise.

**Table 2:** The association between residential environment and arthritis in cohort study results (n = 2,511)

Items	Model 1		Model 2		Model 3	
	**HR (95% CI)**	***P*-value**	**HR (95% CI)**	***P*-value**	**HR (95% CI)**	***P*-value**
Water leakage						
*No*	Ref.		Ref.		Ref.	
*Yes*	1.24 (0.92–1.68)	0.155	1.14 (0.84–1.56)	0.406	1.14 (0.84–1.56)	0.406
Musty smell						
*No*	Ref.		Ref.		Ref.	
*Yes*	1.17 (0.84–1.64)	0.346	1.06 (0.75–1.51)	0.734	1.06 (0.75–1.51)	0.734
Fuel						
*Clean fuel*	Ref.		Ref.		Ref.	
*Non-clean fuel*	1.55 (1.19–2.02)	0.001	1.53 (1.14–2.04)	0.005	1.42 (1.05–1.93)	0.023
Building type						
*Low-rise apartment (<4 floors)*	Ref.		Ref.		Ref.	
*High-rise without elevator (≥4 floors)*	0.77 (0.52–1.15)	0.205	0.77 (0.51–1.16)	0.20	0.65 (0.37–1.12)	0.120
*High-rise with elevator (≥4 floors)*	0.16 (0.02–1.08)	0.06	0.14 (0.02–0.99)	0.049	0.12 (0.02–0.86)	0.035
Cohabitant						
Family	Ref.		Ref.		Ref.	
*Alone*	1.16 (0.85–1.58)	0.353)	0.94 (0.64–1.39)	0.752	0.94 (0.64–1.39)	0.749
*Elderly care institutions*	1.92 (0.71–5.21)	0.198	2.22 (0.79–6.23)	0.128	2.81 (0.97–8.14)	0.057
Bedroom						
*Separate bedroom*	Ref.		Ref.		Ref.	
*Non-separate bedroom*	0.88 (0.49–1.57)	0.661	0.94 (0.52–1.69)	0.831	1.08 (0.59–1.98)	0.804

CI – confidence interval, HR – hazard ratio, ref – reference

### Cross-sectional study

Among 13,886 participants aged ≥65 years, 1,593 (11.5%) had arthritis (**Table 3**). The median age of those was 85.0 years (IQR = 76.0–95.0), and the majority were female (55.9%). Similarly, the arthritis group had a higher proportion of females than the no-arthritis group (64.7% *vs.* 54.7%). When comparing the frequency of window ventilation in summer, participants who ventilated their windows one to five times per week (OR = 0.70; 95% CI = 0.50–0.97) and >5 times per week (OR = 0.66; 95% CI = 0.48–0.90) had a lower risk of arthritis compared to those who did not ventilate at all (**Figure 2**, Panel B; Table S6 in the **Online Supplementary Document**). Additionally, living more than 300 m away from the main road was associated with a decreased risk of arthritis (OR = 0.79; 95% CI = 0.69–0.89).

**Table 3:** Basic characteristics of the cross-sectional study population stratified by arthritis*

Characteristics	All (n = 13,886)	Without arthritis (n = 12,293)	Arthritis (n = 1,593)
Age in years, Mdn (IQR)	85.0 (76.0–95.0)	85.0 (76.0–95.0)	83.0 (75.0–92.0)
Gender (female)	7,756 (55.9)	6,726 (54.7)	1,030 (64.7)
Marital status			
*Divorce/widowed*	7,945 (57.8)	7,075 (58.1)	870 (55.0)
*Married*	5,687 (41.4)	4,993 (41.0)	694 (43.9)
*Unmarried*	117 (0.85)	100 (0.82)	17 (1.08)
Income in CNY per year			
*<10,000*	3,476 (27.4)	3,060 (27.2)	416 (28.7)
*<50,000*	4,319 (34.0)	3,908 (34.8)	411 (28.3)
*≥50,000*	4,898 (38.6)	4,273 (38.0)	625 (43.0)
School years, Mdn (IQR)	1.00 (0.00–6.00)	0.00 (0.00–6.00)	2.00 (0.00–6.00)
Residential area			
*City*	3,056 (22.0)	2,528 (20.6)	528 (33.1)
*Town*	4,632 (33.4)	4,196 (34.1)	436 (27.4)
*Rural*	6,198 (44.6)	5,569 (45.3)	629 (39.5)
Ethnicity (Han)	11,255 (94.2)	9,920 (94.1)	1,335 (95.2)
House ownership (owner)	12,420 (92.7)	11,047 (93.0)	1,373 (90.3)
Cohabitant			
*Living with family*	11,066 (80.7)	9,859 (81.3)	1,207 (76.7)
*Alone*	2,165 (15.8)	1,868 (15.4)	297 (18.9)
*Old people institution*	477 (3.4)	407 (3.3)	70 (4.4)
Own bedroom (yes)	12,567 (94.1)	11,148 (94.1)	1,419 (93.5)
Building type			
*Low rise*	10,740 (80.2)	9,590 (80.8)	1,150 (75.7%)
*High rise without elevator*	1,883 (14.1)	1,624 (13.7)	259 (17.0%)
*High rise with elevator*	772 (5.76)	661 (5.57)	111 (7.30)
Water leakage (yes)	2,528 (19.0)	2,181 (18.4)	347 (23.0)
Musty smell (yes)	1,880 (14.1)	1,637 (13.8)	243 (16.0)
Fuel (clean)	9,864 (73.6)	8,703 (73.2)	1,161 (76.3)
Window ventilation (summer)			
*Don’t open*	399 (2.91)	342 (2.82)	57 (3.67)
*≤5/week*	3,044 (22.2)	2,694 (22.2)	350 (22.6)
*>5/week*	10,249 (74.9)	9,104 (75.0)	1,145 (73.8)
Window ventilation (winter)			
*Don’t open*	2,848 (20.9)	2,548 (21.1)	300 (19.3)
*≤5/week*	5,705 (41.8)	5,056 (41.8)	649 (41.8)
*>5/week*	5,099 (37.3)	4,497 (37.2)	602 (38.8)
Distance from the main road in meters			
*<101*	4,298 (33.6)	3,723 (32.8)	575 (39.7)
*<301*	2,484 (19.4)	2,179 (19.2)	305 (21.0)
*≥301*	6,008 (47.0)	5,438 (48.0)	570 (39.3)
Using insecticide (yes)	1,868 (13.6)	1,646 (13.5)	222 (14.2)
Using repellent (yes)	4,675 (34.1)	4,145 (34.1)	530 (34.0)
Using toilet cleaner (yes)	2,748 (20.0)	2,371 (19.5)	377 (24.2)

IQR – interquartile range, Mdn – median

*Presented as n (%) unless specified otherwise.

### Sensitivity analysis

In the cross-sectional study with 8185 complete cases, the association between summer window ventilation and arthritis disappeared compared with the main results. However, winter window ventilation and insecticide use were associated with the outcome (Table S7 in the **Online Supplementary Document**).

## DISCUSSION

The study reveals significant associations between residential environmental factors and the risk of developing arthritis. By leveraging both cohort and cross-sectional data, we provide a comprehensive analysis of the association between various residential elements and arthritis risk.

In the cohort study, we identified notable risk factors related to fuel type and building characteristics. Specifically, the use of non-clean fuel was associated with a 42% increase in the risk of arthritis (HR = 1.42) compared with clean fuel. This finding is consistent with previous research indicating that indoor air pollution from non-clean fuels, such as coal and wood, can exacerbate inflammatory conditions, thereby worsening arthritis symptoms [27–29]. However, this association should be interpreted with caution because socioeconomic status may partially explain the observed relationship. Non-clean fuel use is often more common among individuals with lower socioeconomic status, who may also experience poorer housing conditions, heavier physical work, reduced access to healthcare, and other arthritis-related risk factors. It is also worth noting that water leakage and musty smell were not significantly associated with incident arthritis in our study, despite the well-established links between dampness, mould exposure, and respiratory or inflammatory diseases [30]. This null finding may suggest that dampness-related exposures have a weaker or less direct effect on arthritis than on respiratory outcomes. Alternatively, self-reported indicators such as water leakage and musty smell may not fully capture the intensity, duration, or biological relevance of dampness and mould exposure. Additionally, residing in high-rise buildings with elevators (≥4 floors) was associated with a substantially lower risk of arthritis (HR = 0.12) compared with living in low-rise apartments (<4 floors). This may be due to better ventilation, improved building materials, and reduced exposure to dampness and mould in high-rise buildings [31–33]. On the other hand, no significant association was observed for high-rise buildings without elevators. One possible explanation is that residents in these buildings may need to climb stairs more frequently, which could increase mechanical loading on the joints. However, this finding should be interpreted with caution. The high-rise-with-elevator category included only 41 participants, among whom only two incident arthritis cases were observed during follow-up. Such sparse data may lead to unstable effect estimates and inflated hazard ratios. Therefore, the observed association may reflect statistical uncertainty rather than a true protective effect. Additional studies with larger sample sizes and greater numbers of exposed participants are required to verify this finding.

Our cross-sectional study further clarifies the association between specific residential environments and arthritis. Compared to no ventilation, regular window ventilation during summer is protective against arthritis. Such daily ventilation, one to five times weekly (OR = 0.70) and more than five times weekly (OR = 0.66), was associated with a lower risk of arthritis compared with no ventilation. This would underscore the importance of good indoor air quality and reduced levels of pollutants and moisture. Good indoor air quality, achieved by reducing pollutants and moisture, is also essential in minimising inflammatory responses that can exacerbate conditions like arthritis [34]. Studies have shown that improvements in indoor air quality can significantly impact overall health, particularly for those with chronic inflammatory conditions [35–37].

A meaningful association with arthritis risk was also observed near major roadways. Living in a location more than 300 m away from major roadways was related to a reduction of 21% in the risk of arthritis (OR = 0.79) as compared to living within distances of 0–100 m. This finding demonstrates the negative effects of traffic-related air pollution, including particulate matter and nitrogen dioxide, which can increase inflammation and, in turn, increase the risk of arthritis [14,38,39]. Chronic exposure to traffic noise could be another contributory factor [40,41]. Long-term exposure to high levels of noise pollution must be expected to increase stress and may diminish sleep, both of which exacerbate inflammatory states such as arthritis [42]. Studies have shown that noise pollution can elevate stress hormone levels, such as cortisol, which has pro-inflammatory effects and can worsen joint health [43].

These results imply that the quality of the residential environment needs to be improved to prevent arthritis. The association with non-clean fuels is positive and calls for promoting cleaner cooking and heating technologies, particularly in lower-income settings where these fuels are most commonly used. It was attractive to see the protective impact of high-rise buildings with elevators and that associated with frequent window ventilation. Building design and indoor airspace management were also significant. The correlation between proximity to main roads and the risk of arthritis also suggests that urban planning should prioritise measures that reduce residential exposure to traffic pollutants and noise. Policies that encourage the development of residential areas away from major traffic arteries and enhance green spaces can contribute to better overall health outcomes.

However, this study has several limitations. The arthritis data rely on self-reporting, which may be influenced by recall bias, and the specific subtypes of arthritis cannot be determined through questionnaire surveys. Additionally, participants without follow-up arthritis data were excluded from the cohort analysis, potentially introducing loss-to-follow-up bias. Although most residential environmental exposures were not associated with follow-up status, bedroom number was significantly associated with follow-up participation. Therefore, while substantial selection bias is unlikely for most exposures, findings regarding bedroom number should be interpreted with caution, as residual bias from differential loss to follow-up cannot be completely ruled out. Third, the follow-up period was relatively short for arthritis, which may have limited our ability to capture long-term effects of built-environment exposures and reduced statistical power. Future studies with longer follow-up periods are needed to confirm the observed associations and evaluate potential long-term effects of residential environment exposures on arthritis development. Fourthly, some exposure categories contained relatively small numbers of participants and incident arthritis cases. Consequently, the corresponding hazard ratio estimates may be unstable and should be interpreted cautiously, and additional studies with larger sample sizes and greater numbers of exposed participants are required to verify this finding. Although multiple imputation was used to reduce potential bias due to missing data, this approach assumes that data are missing at random conditional on observed variables. This assumption cannot be fully tested, and the inconsistency between the MICE-based and complete-case analyses suggests that some cross-sectional findings may be influenced by the mechanism of missing data. Finally, the cross-sectional component of this study is inherently susceptible to reverse causation because exposure and outcome were assessed simultaneously. Therefore, the cross-sectional findings should be interpreted primarily as identifying potential environmental factors associated with arthritis rather than establishing causal relationships.

## CONCLUSIONS

These findings highlight the critical need for public health interventions and policies to improve residential environmental quality to mitigate the burden of arthritis, especially in rapidly urbanising and ageing populations. This research not only fills gaps in the current literature but also provides scientific evidence to inform the development of arthritis prevention and control strategies, holding significant theoretical and practical value.

## Additional material


Online Supplementary Document

## Data Availability

**Data availability:** The data used in this study are from the CLHLS. The data set is publicly accessible *via* the Peking University Open Research Data Platform at https://doi.org/10.18170/DVN/WBO7LK. Access requires registration and approval from the CLHLS user group. The data set is described in the repository documentation and is cited in the References section of this manuscript [25].
