# Supplementary material for: The association between the residential environment and arthritis among the elderly in China: evidence from cohort and cross-sectional studies
Source: J Glob Health. 2026 Aug 14;16:04276. doi: 10.7189/jogh.16.04276 (PMC13473932; doi:10.7189/jogh.16.04276)
Supplement: Supplementary file 1 [file jogh-16-04276-s001.pdf]

Supplement to: Xiangbin J, Xiong X, Zichao J, Haoze Y. The association between the residential environment and arthritis among the elderly in China: evidence from cohort and cross-sectional studies. J Glob Health. 2026;16:04276.

Table S1. STROBE Statement—checklist of items that should be included in reports of observational studies

	**Item No.**	**Recommendation**	**Page No.**	**Relevant text from manuscript**
**Title and abstract**	1	(*a*) Indicate the study's design with a commonly used term in the title or the abstract	1	Line 24-32
		(*b*) Provide in the abstract an informative and balanced summary of what was done and what was found	1	Line 33-42
**Introduction**				
Background/rationale	2	Explain the scientific background and rationale for the investigation being reported	2	Line 46-66
Objectives	3	State specific objectives, including any prespecified hypotheses	3	Line 67-75
**Methods**				
Study design	4	Present key elements of study design early in the paper	4	Line 92-104
Setting	5	Describe the setting, locations, and relevant dates, including periods of recruitment, exposure, follow-up, and data collection	3-4	Line 77-91
Participants	6	(*a*) *Cohort study*—Give the eligibility criteria, and the sources and methods of selection of participants. Describe methods of follow-up<br>*Case-control study*—Give the eligibility criteria, and the sources and methods of case ascertainment and control selection. Give the rationale for the choice of cases and controls<br>*Cross-sectional study*—Give the eligibility criteria, and the sources and methods of selection of participants	3-4	Line 77-91
		(*b*) *Cohort study*—For matched studies, give matching criteria and number of exposed and	3-4	Line 77-91

		unexposed<br>*Case-control study*—For matched studies, give matching criteria and the number of controls per case		
Variables	7	Clearly define all outcomes, exposures, predictors, potential confounders, and effect modifiers. Give diagnostic criteria, if applicable	4-5	Line 92-112
Data sources/ measurement	8*	For each variable of interest, give sources of data and details of methods of assessment (measurement). Describe comparability of assessment methods if there is more than one group	3	Line 77-81
Bias	9	Describe any efforts to address potential sources of bias	4-5	Line 105-112
Study size	10	Explain how the study size was arrived at	3-4	Line 82-91

Continued on next page

Quantitative variables	11	Explain how quantitative variables were handled in the analyses. If applicable, describe which groupings were chosen and why	4	Line 92-104
Statistical methods	12	(*a*) Describe all statistical methods, including those used to control for confounding	5-6	Line 113-148
		(*b*) Describe any methods used to examine subgroups and interactions	5-6	Line 113-148
		(*c*) Explain how missing data were addressed	5-6	Line 113-148
		(*d*) *Cohort study*—If applicable, explain how loss to follow-up was addressed<br>*Case-control study*—If applicable, explain how matching of cases and controls was addressed<br>*Cross-sectional study*—If applicable, describe analytical methods taking account of sampling strategy	5-6	Line 113-148
		(*e*) Describe any sensitivity analyses	6	Line 149-151
**Results**				
Participants	13*	(a) Report numbers of individuals at each stage of study—eg numbers potentially eligible, examined for eligibility, confirmed eligible, included in the study, completing follow-up, and analysed	3-4	Line 82-91
		(b) Give reasons for non-participation at each stage	3-4	Line 82-91
		(c) Consider use of a flow diagram	4	Line 89
Descriptive data	14*	(a) Give characteristics of study participants (eg demographic, clinical, social) and information on exposures and potential confounders	6-7	Line 153-169
		(b) Indicate number of participants with missing data for each variable of interest	6-7	Line 153-169
		(c) *Cohort study*—Summarise follow-up time (eg, average and total amount)	6	Line 153-160
Outcome data	15*	*Cohort study*—Report numbers of outcome events or summary measures over time	6	Line 153-160
		Case-control study—Report numbers in each exposure category, or summary measures of exposure		
		Cross-sectional study—Report numbers of outcome events or summary measures	7	Line 161-169
Main results	16	(*a*) Give unadjusted estimates and, if applicable, confounder-adjusted estimates and their precision (eg, 95% confidence interval). Make clear which confounders were adjusted for and why they were included	6-7	Line 153-169
		(*b*) Report category boundaries when continuous variables were categorized	6-7	Line 153-169

		(*c*) If relevant, consider translating estimates of relative risk into absolute risk for a meaningful time period	6-7	Line 153-169
Other analyses	17	Report other analyses done—eg analyses of subgroups and interactions, and sensitivity analyses	7	Line 170-175
**Discussion**				
Key results	18	Summarise key results with reference to study objectives	7-10	Line 176-229
Limitations	19	Discuss limitations of the study, taking into account sources of potential bias or imprecision. Discuss both direction and magnitude of any potential bias	10-11	Line 236-259
Interpretation	20	Give a cautious overall interpretation of results considering objectives, limitations, multiplicity of analyses, results from similar studies, and other relevant evidence	7-11	Line 176-259
Generalisability	21	Discuss the generalisability (external validity) of the study results	7-11	Line 176-259
**Other information**				
Funding	22	Give the source of funding and the role of the funders for the present study and, if applicable, for the original study on which the present article is based	11	Line 266-268

*Give information separately for cases and controls in case-control studies and, if applicable, for exposed and unexposed groups in cohort and cross-sectional studies.

Note: An Explanation and Elaboration article discusses each checklist item and gives methodological background and published examples of transparent reporting. The STROBE checklist is best used in conjunction with this article (freely available on the Web sites of PLoS Medicine at http://www.plosmedicine.org/, Annals of Internal Medicine at http://www.annals.org/, and Epidemiology at http://www.epidem.com/). Information on the STROBE Initiative is available at www.strobe-statement.org.

Table S2. Outline of JoGH's Guidelines for Reporting Analyses of Big Data Repositories Open to the Public (GRABDROP) items

**JoGH guideline item**	**Answer**
1. Please list all papers published by each co-author in previous three years that were based on secondary analysis of a big data repository	1.Long X, Chen R, Lu Y, Ma B, Chang Y, **Jiang Z**, Lei T. Comprehensive evaluation of appendicular lean mass and sarcopenia on human health: evidence from the NHANES program. Exp Gerontol. 2026 Mar;215:113049. doi: 10.1016/j.exger.2026.113049. Epub 2026 Jan 22. PMID: 41579911.<br>2.Xu Z, Luo X, Diao W, Tang X, Zhang Y, Wang J, **Jiang Z**, Lei T. Contributing effects of sarcopenia on cancer occurrence: novel evidence based on NHANES 1999-2020 and two-sample mendelian randomization study. Oncologist. 2025 Nov 11;30(11):oyaf369. doi: 10.1093/oncolo/oyaf369. PMID: 41206069; PMCID: PMC12628308.<br>3.Long Y, Pan Y, Yang H, Wang X, **Jiang Z**, Sun T. The Genetic Causal Effect of Autoimmune Diseases on pan-Cancers: Evidence from Mendelian Randomization. J Cancer. 2025 Jan 1;16(2):567-576. doi: 10.7150/jca.103693. PMID: 39744497; PMCID: PMC11685688.<br>4.Wang Y, **Jiang Z**, Lei J, Tan Z, Wu Y, Hu Y, Yang J, Li M, Lei T, Hua L. Correlation of volatile organic chemical exposure with uric acid metabolism among US population: A cross-sectional study from NHANES program. J Environ Sci (China). 2025 Jun;152:611-622. doi: 10.1016/j.jes.2024.04.002. Epub 2024 Apr 11. PMID: 39617581.<br>5.Lei T, **Jiang Z**, Wang J, Nan J, Hua L, Zhu Z, Hu Y. Genetic Influence of the Brain on Muscle Structure: A Mendelian Randomization Study of Sarcopenia. J Cachexia Sarcopenia Muscle. 2025 Feb;16(1):e13647. doi: 10.1002/jcsm.13647. Epub 2024 Nov 13. PMID: 39535371; PMCID: PMC11695463.<br>6.Zhang Y, **Jiang Z**, Chen L, Lei T, Zheng X. Repurposing lipid-lowering drugs on asthma and lung function: evidence from a genetic association analysis. J Transl Med. 2024 Jul 3;22(1):615. doi: 10.1186/s12967-024-05359-5. PMID: 38961500; PMCID: PMC11223406.

JoGH guideline item	Answer
	7.Lei T, **Jiang Z**, Qian H, Backstein D, Lei P, Hu Y. Comparison of Single-Radius with Multiple-Radius Femur in Total Knee Arthroplasty: A Meta-Analysis of Prospective Randomized Controlled Trials. Orthop Surg. 2022 Sep;14(9):2085-2095. doi: 10.1111/os.13391. Epub 2022 Aug 4. PMID: 35924690; PMCID: PMC9483041.
2. Please explain the key elements of your study design and the use of the available datasets that make your study an original scientific contribution	This study combined complementary cohort and cross-sectional analyses using the Chinese Longitudinal Healthy Longevity Survey (CLHLS), a large, publicly accessible, population-based survey of adults aged 65 years and older from 23 Chinese provinces. The cohort analysis linked residential-environment exposures measured in 2014 to incident arthritis in 2018 (n=2,511), whereas the cross-sectional analysis evaluated additional residential factors that were available only in the 2018 wave (n=13,886). Twelve residential factors covering indoor air quality, housing conditions, household practices, building characteristics, and proximity to major roads were examined. Cox proportional hazards models were used for the cohort analysis and logistic regression for the cross-sectional analysis, with exposure-specific confounder adjustment, multiple imputation for missing covariates, and complete-case sensitivity analyses. The original contribution is the integrated evaluation of a broad range of residential environmental factors in relation to arthritis among older Chinese adults, including prospective evidence for factors available across waves and complementary evidence for newly measured 2018 exposures.
3. Please list all publications that addressed similar research questions in the same dataset and indicate where you cited them in your paper	The manuscript cites the following publications based on the CLHLS in the Methods section under "Study population": Zhong J, Chen L, Li C, et al. Association of lifestyles and multimorbidity with mortality among individuals aged 60 years or older: Two prospective cohort studies. SSM Popul Health. 2024;26:101673 [reference 22]; and Yu L, Yang M, Ye KX, et al. Investigating the impact of tea consumption on cognitive function and exploring tea-genetic interactions in older adults aged 65–105 years: Findings from the 2002–2018 CLHLS data. J Prev Alzheimers Dis. 2024;11:769–779 [reference 23]. The CLHLS dataset and repository documentation are also cited in "Study population" as references 25 and in the Data availability statement as reference 25. These prior CLHLS studies address other health outcomes and do not duplicate the present study's combined cohort and cross-sectional assessment of residential environmental factors and arthritis.

JoGH guideline item	Answer
4. Please explain how you addressed multiple testing through an appropriately rigorous statistical threshold and indicate this in the methods section	No formal multiplicity correction was applied because the analyses were exposure-specific and exploratory. Statistical significance was evaluated using two-sided $P<0.05$, and the interpretation emphasized adjusted effect estimates with 95% confidence intervals rather than P values alone. Each residential factor was assessed in a separately specified model, with Model 3 adjusted for its prespecified potential confounders and treated as the primary model. Robustness was further examined in complete-case sensitivity analyses. Given the number of exposures evaluated, statistically significant findings should be interpreted cautiously and require confirmation in independent datasets. This clarification should be included in the Methods subsection “Multivariate Cox and logistic regression analysis.”
5. Please declare to what extent have AI chatbots been used in developing your paper and to which parts of the paper did they contribute	No AI chatbots were used in the study design, data collection, statistical analysis, interpretation of the results. AI used for polishing the manuscript.

Table S3. Missing data of cohort study

Variable	Missing Data	
	N	%
Age	0	0
Gender	0	0
Marital status	35	1.39
Income	157	6.25
School years	29	1.15
Residential area	0	0

Ethnicity	288	11.47
House ownership	20	0.80
Cohabitant	24	0.96
Bedroom	27	1.08
Building Type	28	1.12
Water leakage	32	1.27
Musty smell	20	0.80
Fuel	20	0.80
Arthritis	0	0
Follow-up time	0	0

Table S4. Missing data of Cross-sectional study

**Variable**	**Missing Data**	
	**N**	**%**
Age	0	0
Gender	0	0
Marital status	136	0.98
Income	1,190	8.59
School years	2,078	15.01
Residential area	0	0
Ethnicity	1,940	14.01
House ownership	480	3.47

Cohabitant	178	1.29
Bedroom	524	3.78
Building Type	488	3.52
Water leakage	550	3.97
Musty smell	509	3.68
Window Ventilation (Summer)	193	1.39
Window Ventilation (Winter)	230	1.66
Distance from the main road	1,088	7.86
Using insecticide	171	1.23
Using repellent	162	1.17
Using toilet cleaner	169	1.22
Arthritis	0	0

Table S5. Details on model 3 for the cohort and cross-sectional studies.

Exposures	Model 3
**Cohort study (Cox regression)**	
Musty smell	Arthritis ~ musty smell+ water leakage+ age+ gender+ ethnicity+ income+ school years+ marital status+ cohabitant+ residential area+ building type
Water leakage	Arthritis ~ water leakage+ musty smell+ age+ gender+ ethnicity+ income+ school years+ marital status+ cohabitant+ residential area+ building type
Building type	Arthritis ~ building type + age+ gender+ ethnicity+ income+ school years+ marital status+ residential area
Cohabitant	Arthritis ~ cohabitant+ age+ gender+ ethnicity+ income+ school years+ marital status+ residential area+ building type

Fuel	Arthritis ~ fuel+ age+ gender+ ethnicity+ income+ school years+ marital status+ cohabitant+ residential area+ building type
Bedroom	Arthritis ~ bedroom+ age+ gender+ ethnicity+ income+ school years+ marital status+ cohabitant+ residential area+ building type
**Cross-sectional study (logistic regression)**	
Window ventilation in summer	Arthritis ~ window ventilation in summer+ window ventilation in winter+ age+ gender+ ethnicity+ income+ school years+ marital status+ cohabitant+ residential area+ building type+ musty smell+ water leakage + using insecticide+ using repellent+ using toilet cleaner+ fuel+ distance from the main road
Window ventilation in winter	Arthritis ~ window ventilation in winter+ window ventilation in summer+ age+ gender+ ethnicity+ income+ school years+ marital status+ cohabitant+ residential area+ building type+ musty smell+ water leakage + using insecticide+ using repellent+ using toilet cleaner+ fuel+ distance from the main road
Using insecticide	Arthritis ~ using insecticide+ using repellent+ using toilet cleaner+ age+ gender+ ethnicity+ income+ school years+ marital status+ cohabitant+ residential area+ building type+ fuel+ distance from the main road
Using repellent	Arthritis ~ using repellent+ using insecticide+ using toilet cleaner+ age+ gender+ ethnicity+ income+ school years+ marital status+ cohabitant+ residential area+ building type+ fuel+ distance from the main road
Using toilet cleaner	Arthritis ~ using toilet cleaner+ using insecticide+ using repellent+ age+ gender+ ethnicity+ income+ school years+ marital status+ cohabitant+ residential area+ building type+ fuel+ distance from the main road
Distance from the main road	Arthritis ~ distance from the main road+ age+ gender+ ethnicity+ income+ school years+ marital status+ residential area+ building type

Table S6. The association between residential environment and arthritis in Cross-Sectional study results (N=13,886).

	Model 1	Model 2	Model 3

	OR	95%CI	*P*	OR	95%CI	*P*	OR	95%CI	*P*
**Window Ventilation (Summer)**									
0 time/week	Reference								
1-5 times/week	0.78	0.57–1.05	0.103	0.74	0.55–1.01	0.059	0.7	0.50–0.97	**0.032**
More than 5 times/week	0.75	0.57–1.01	0.055	0.69	0.52–0.93	**0.014**	0.66	0.48–0.90	**0.009**
**Window Ventilation (Winter)**									
0 times/week	Reference								
1-5 times/week	1.10	0.95–1.27	0.212	1.03	0.89–1.19	0.725	1.08	0.92–1.27	0.328
> 5 times/week	1.15	0.99–1.33	0.059	1.06	0.91–1.23	0.491	1.1	0.93–1.31	0.26
**Distance from the main road**									
0-100 meters	Reference								
101-300 meters	0.91	0.78–1.05	0.202	0.90	0.77–1.05	0.169	0.89	0.76–1.03	0.122
> 300 meters	0.68	0.61–0.77	**<0.001**	0.72	0.64–0.82	**<0.001**	0.79	0.69–0.89	**<0.001**

**Using insecticide**									
NO	Reference								
YES	1.06	0.91–1.23	0.467	1.09	0.93–1.27	0.274	1.10	0.93–1.30	0.266
**Using repellent**									
NO	Reference								
YES	0.99	0.89–1.11	0.930	1.03	0.92–1.15	0.669	1.08	0.95–1.22	0.245
**Using toilet cleaner**									
NO	Reference								
YES	1.32	1.17–1.50	**<0.001**	1.21	1.06–1.38	**0.004**	1.12	0.97–1.29	0.112

Abbreviations: OR, odds ratio; CI, confidence interval.

Table S7. Sensitive analysis of the association between residential environment and arthritis from cohort study (N=1,976).

	**Model 1**			**Model 2**			**Model 3**		
	**HR**	**95%CI**	***P***	**HR**	**95%CI**	***P***	**HR**	**95%CI**	***P***

**Water leakage**									
NO	Reference								
YES	1.22	0.87–1.73	0.253	1.15	0.81–1.63	0.446	1.11	0.74–1.66	0.611
**Musty smell**									
NO	Reference								
YES	1.18	0.80–1.72	0.404	1.11	0.75–1.63	0.615	1.03	0.66–1.61	0.900
**Fuel**									
Highly polluting fuel	Reference								
Non high polluting fuel	1.55	1.15–2.09	**0.004**	1.63	1.18–2.27	**0.003**	1.56	1.10–2.21	**0.012**
**Building type**									
Low-rise apartment (<4 floors)	Reference								
High-rise without elevator (≥4 floors)	0.86	0.57–1.30	0.465	0.79	0.51–1.23	0.299	0.63	0.35–1.13	0.122
High-rise with elevator (≥ 4 floors)	0.14	0.02–0.99	**0.049**	0.11	0.01–0.79	**0.029**	0.09	0.01–0.67	**0.019**
**Cohabitant**									
Family	Reference								
Alone	1.01	0.69–1.46	0.979	0.79	0.51–1.24	0.311	0.79	0.50–1.24	0.298
Elderly care institutions*	0	0.00–Inf	0.994	0	0.00–Inf	0.994	0	0.00–Inf	0.994
**Bedroom**									
Separate bedroom	Reference								
Non-Separate bedroom	0.79	0.43–1.46	0.455	0.86	0.46–1.59	0.63	1.02	0.54–1.91	0.951

Abbreviations: HR, hazard ratio; CI, Confidence interval.

* None of the participants (n=3) in this variable have arthritis.

Table S8. Sensitive analysis of the association between residential environment and arthritis from cross-sectional study (N=8,185).

	**Model 1**			**Model 2**			**Model 3**		
	**OR**	**95%CI**	***P***	**OR**	**95%CI**	***P***	**OR**	**95%CI**	***P***
**Window Ventilation (Summer)**									
0 time/week	Reference								
1-5 times/week	1.14	0.73–1.87	0.572	1.06	0.67–1.74	0.819	0.87	0.54–1.47	0.601
More than 5 times/week	0.92	0.60–1.48	0.700	0.82	0.53–1.33	0.391	0.72	0.45–1.21	0.196
**Window Ventilation (Winter)**									
0 times/week	Reference								
1-5 times/week	1.33	1.09–1.63	**0.005**	1.23	1.00–1.51	0.053	1.25	1.01–1.57	**0.042**
More than 5 times/week	1.21	0.99–1.49	0.072	1.08	0.88–1.34	0.477	1.19	0.94–1.50	0.150
**Distance from the main road**									
0-100 meters	Reference								
101-300 meters	0.78	0.65–0.95	**0.012**	0.78	0.64–0.95	**0.012**	0.77	0.63–0.93	**0.008**
more than 300 meters	0.69	0.59–0.80	**<0.001**	0.74	0.63–0.87	**<0.001**	0.83	0.71–0.98	**0.026**
**Using insecticide**									
NO	Reference								
YES	1.16	0.95–1.40	0.135	1.2	0.98–1.46	0.065	1.33	1.07–1.66	**0.010**
**Using repellent**									
NO	Reference								
YES	0.88	0.76–1.02	0.096	0.93	0.80–1.08	0.320	0.94	0.79–1.11	0.445
**Using toilet cleaner**									
NO	Reference								
YES	1.2	1.02–1.40	**0.030**	1.08	0.91–1.28	0.360	0.99	0.83–1.19	0.954

Abbreviations: OR, odds ratio; CI, confidence interval.
